# Transcriptomic Analysis of circRNAs in the Peripheral Blood of Nonarteritic Anterior Ischemic Optic Neuropathy

**DOI:** 10.1155/2020/5732124

**Published:** 2020-11-26

**Authors:** Jinshan Suo, Xinling Xu, Haoyan Xu, Naifang Hou, Jiaming Zhang, XIao Han, Yanwen Zheng, Xinling Wang, Xiao Han

**Affiliations:** Department of Ophthalmology, The Fourth Affiliated Hospital of China Medical University, Key Laboratory of Lens Research of Liaoning Province, Eye Hospital of China Medical University, Shenyang, 110005 Liaoning Province, China

## Abstract

The aim of the study is to explore the expression profile variation of circular RNAs (circRNAs) in the peripheral blood of subjects with nonarteritic anterior ischemic optic neuropathy (NAION) and without NAION, to analyze the differential expression results, and to predict the role of circRNAs in disease development, providing novel ideas and methods for treatment and diagnosis. High-throughput sequencing to explore the expression profiles of RNAs in the peripheral blood of 6 NAION patients and 5 healthy controls was applied. Quality control obtained the advanced data from the original data by ticking out the unqualified data. Then, cluster analysis, volcano plot, coexpression network, and protein-protein interaction network (PPI) were performed. Gene ontology (GO) analysis and Kyoto Encyclopedia of Genes and Genomes (KEGG) pathway were used to analyze the whole expressed genes. Lastly, the quantitative real-time Polymerase Chain Reaction (qRT-PCR) was used to verify those significantly differentially expressed circRNAs and do some bioinformatics analysis and prediction in 12 NAION patients and 12 controls. There were significant differences in the expression of 49 circRNAs in the peripheral blood of NAION patients, in which there were 24 upregulations and 25 downregulations (variation folds > 2 and *P* < 0.05), and it was confirmed that hsa_circ_0005583, hsa_circ_0003922, hsa_circ_0002021, and hsa_circ_0000462 were significantly downregulated (variation folds > 2 and *P* < 0.05), especially hsa_circ_0005583 which was the most significantly changed one (*P* < 0.001), and are related to processes such as neurodegeneration, oxidative stress, immunity, and metabolism. The expression profile of circRNAs in the peripheral blood of NAION patients is significantly changed, enriching our understanding of the disease.

## 1. Introduction

Nonarteritic anterior ischemic optic neuropathy (NAION) is more common in the middle-aged and elderly population, especially is the most common acute optic neuropathy in adults over 50 years of age [1]. Patients typically present with acute painless monocular vision loss and typical visual field defects associated with optic nerve head (ONH) edema [2, 3]. The pathogenesis of NAION remains unknown, and many epidemiological risk factors including diabetes mellitus [4, 5], systemic hypertension, acute hypovolemia [1, 6], elevated cholesterol levels [7], obstructive sleep apnea [8–11], and homocysteinemia [11] are associated with the pathogenesis of NAION. In addition, polymorphisms such as Endothelin-1 (ET-1), Angiotensin-Converting Enzyme (ACE), and Atonal Homolog7 (ATOH7) increase the susceptibility of NAION [12]. At the same time, the decrease of blood flow in the short posterior ciliary artery found by Doppler ultrasonography indicates that microvascular ischemia also plays a role in the pathogenesis of NAION [13]. Although the exact pathogenesis of NAION is unclear, researchers now believe that NAION is a multifactorial copromoting neuropathy. It is generally accepted that the onset of NAION mainly results from a brief interruption of the ONH circulation caused by hypo- or nonperfusion of the short posterior ciliary artery [3]. At present, there is no clinically proven effective drug for NAION, and further research on its pathogenesis and treatment is urgently needed.

Circular RNAs (circRNAs) are a new class of endogenous noncoding RNAs, without 5′ cap and 3′ tail structures of linear RNAs, characterized by a covalently closed-loop structure formed by a back-splicing event [14]. circRNAs widely exist in eukaryotic cells. Researchers have found that the expression of circRNAs is related to the occurrence and development of many diseases and has an important role to play in various cancers, cardiovascular diseases, and neurological diseases [15–17]. Recently, researchers have found that circRNAs express and play an important role in many ocular tissues and ocular diseases, such as “sponge molecular” miRNA regulating the expression of target genes in retinoblastoma [18], diabetic retinopathy [18, 19], pterygium [20], and neuromyelitis optica [21]. At present, the treatment of NAION includes systemic or topical corticosteroid treatment [22, 23] to improve microcirculation, neurotrophic therapy, and acupuncture [1]. But these treatments have different effects. To further explore the pathogenesis and prognosis of NAION, the researchers have found some abnormal coagulation factors; ATOH7 TT genotype carriers conferred a significantly increased risk of NAION [12]. One study reported that administration of recombinant human granulocyte colony-stimulating factor (G-CSF) is neuroprotective in the rat model of anterior ischemic optic neuropathy, as demonstrated both structurally by Retinal Ganglion Cell (RGC) density and functionally by flash visual-evoked potentials (FVEP) [24], and two cases have been reported that one copy of the variable number tandem repeat (VNTR) B alleles of the GPIb*α* gene increases the risk of NAION and the second eye involvement [25, 26], neurological factors, and factors related to NAION gene expression may be associated to the occurrence and development of NAION. A comprehensive and detailed study of the epigenetic and molecular basis of NAION is the key to early diagnosis, optimal treatment, and prognosis. Therefore, this study used high-throughput sequencing technology to determine the expression profiles of circRNAs in the peripheral blood of patients with NAION and predicted their functions and mechanisms through recognized bioinformatics analysis technology. Maybe we can find some biological markers related to the pathogenesis and prognosis of NAION, which provides new ideas for the study of the etiology, efficacy, and prognosis of NAION and provides a more optimized treatment to improve the visual quality of patients and the quality of life.

## 2. Materials and Methods

### 2.1. Subjects and Groups

This study collected peripheral venous blood samples from 18 patients with nonarteritic anterior ischemic optic neuropathy (NAION) (*n* = 18) (male/female: 9/9) who visited the Eye Center of the Fourth Affiliated Hospital of China Medical University from September 2017 to May 2018 and 17 recruited healthy controls matched with age and gender (control) (*n* = 17) (male/female: 8/9). The demographic and clinical features of all the patients and healthy controls are summarized in Table 1.

The inclusion criteria for patients with NAION were as follows: (1) conforms to the diagnosis of NAION [2]: sudden, monocular acute painless visual loss; characteristic visual field defect associated with physiological blind spot; monocular relative afferent pupillary disorder; acute orbital optic disc localization or diffuse edema, unclear borders, with or without linear hemorrhage around the nipple; fundus fluorescein angiography manifested as early papillary localization or diffuse filling defect or delay, or at the late-stage, the leak in the optic disc is strongly fluorescent and all subjects without treated and with an acute phase of <2 weeks; (2) clinical test: blood glucose value: 3.9~6.1 mmol/L; glycated hemoglobin (HbA1c): 4%~6%; total cholesterol: 2.8 ~6.5 mmol/L; triglyceride: 0.56~1.70 methyl/L; high-density lipoprotein: 1.03~2.07 mmol/L; low-density lipoprotein 1.0~4.4 mmol/L; blood pressure: 90~139/60~80 mmHg.

The exclusion criteria for the NAION group were that NAION patients with the duration > 2 weeks or other eye diseases; ischemic heart disease, congenital heart disease, coronary artery disease, hypercholesterolemia, hyperlipidemia, hypertension, diabetes, rheumatoid, thyroid hyperfunction, myelitis, and diseases of the blood system are also excluded.

### 2.2. Extract RNAs

According to the procedure, total RNAs were extracted from the peripheral blood of the subjects with TRIzol reagent (Invitrogen, Grand Island, New York, USA). The RNA extracted from the samples meets the standards of OD A260/A280 (>1.8) and A260/A230 (>1.6) standards (Tray for 16 Spectrophotometer cells, Agilent, Agilent Technologies, Agilent, USA). The total quantity and quality of RNA extracted were evaluated by the Agilent 2100 Bioanalyzer (Agilent, Agilent Technologies, California, USA) and RNA 6000 Nano LabChip Kit (Agilent, Agilent Technologies, California USA), and the RNA integrity score (RIN) is >7.0.

### 2.3. Sequencing and Analysis

In this experiment, peripheral blood RNA of 6 NAION patients and 5 control subjects were sequenced by Beijing Yuanyi Gene Technology Co., Ltd. (Beijing, China). The total transcriptome library was constructed according to the Ribo-Zero Magnetic Gold Kit (Illumina, Illumina, Inc., San Diego, USA) and Illumina's NEBNext® UltraTM RNA Library Preparation Kit (NEB, New England Biolabs, Ipswich, MA, USA) operating instructions. The Bioanalyzer 2100 system (Agilent, Agilent Technologies, California, USA) and qRT-PCR (Kapa, Kapa Biosystems, Cape Town, South Africa) were used to control the quality and quantification of the library. The resulting library was initially sequenced on the HiSeq X instrument (Illumina, Illumina, Inc., San Diego, USA), which produces paired-end readings of 150 nucleotides. Sequence reads were matched to the human genome (GRCh38) using the TopHat 2.0 program [27], and the resulting alignment files were reconstructed with Cufflinks. RefSeq (https://www.ncbi.nlm.nih.gov/protein/) and circBase (http://www.circbase.org/) transcript databases were selected as annotations for the analysis of mRNAs and circRNAs. Applied to predict circRNAs, CIRCexplorer [28] (https://circexplorer2.readthedocs.io/en/latest/) standardizes the reading counts of each transcript as the length of a single transcript and the total mapping reading counts in each sample and expresses them as FPKM. The log2 (multiple changes) absolute values > 1 and *P* < 0.05 between the two samples were used to identify differentially expressed genes and transcripts [27, 28].

### 2.4. Quantitative Real-Time Polymerase Chain Reaction (qRT-PCR) Verification

RNAs were extracted from blood samples of the remaining 12 NAION individuals and 12 control individuals for qRT-PCR verification. cDNA was synthesized using MMLV reverse transcriptase (Invitrogen, Invitrogen Corporation, California, USA) according to the instructions. qRT-PCR was then performed in a 20 *μ*L reaction system, including the SYBR Green Master Mix (Thermo Fisher Technology Company, San Jose, CA, USA) 10 *μ*L, PCR forward primer 0.8 *μ*L (10 *μ*M), PCR reverse primer 0.8 *μ*L (10 *μ*M), ROX 0.4 *μ*L, cDNA 2 *μ*L, and enzyme-free water (Invitrogen, Carlsbad, CA, USA) 6 *μ*L. The reaction system began to react at 95°C for 5 minutes and then reacted at 95°C (5 seconds) and 60°C (34 seconds) for a total of 40 cycles. The reaction was carried out in three separate wells with *β*-actin (Invitrogen, Carlsbad, CA, USA) as an internal reference, and the relative RNA expression level was calculated using the *Δ*Ct value and the 2-*ΔΔ*Ct value [29]. *T*-test was performed to show that when *P* < 0.05, the difference was statistically significant, and the values were expressed as M ± SD. We randomly selected 10 differentially expressed circRNAs from the sequencing group for validation (change fold > 2 and *P* < 0.05), including 3 upregulated circRNAs (hsa_circ_0001723, hsa_circ_0000462, and hsa_circ_0001811) and seven downregulated circRNAs (hsa_circ_0003922, hsa_circ_0066536, hsa_circ_0005583, hsa_circ_0001626, hsa_circ_0000339, hsa_circ_0023694, and hsa_circ_0002021). The results of 10 gene primer sequences are shown in Table 2.

### 2.5. Coexpression Network and PPI Network and Module Analysis

We applied the Pearson correlation index to calculate the correlation between coexpression analysis and statistical significance to construct a circRNA-mRNA coexpression network, in which the coefficient parameter values were >0.99 or <-0.99, and further analysis of significant differences in RNAs when *P* < 0.001. The PPI network is predicted using a search tool that searches for interacting genes online (STRING; http://string-db.org) (version 11.0) [30]. In the interaction network, the combined score > 0.4 is considered to have significant statistical significance. The PPI interaction network diagram was then further modified using Cytoscape (version 3.7.1) software [31]. Cytoscape is an open bioinformatics software platform for visualizing molecular interaction networks. Cytoscape's plug-in molecular complex detection (MCODE) (version 1.4.2) is used to cluster a given network based on the topology to find densely connected regions. Use Cytoscape to draw a PPI network and use MCODE to identify the most important modules in the PPI network. The selection criteria are as follows: MCODE score > 5, degree cutoff = 2, node score cutoff = 0.2, maximum depth = 100, and *k* score = 2.

### 2.6. GO and KEGG Pathway Analysis

We describe genes and gene products of cell components, molecular functions, and biological processes through Gene Ontology (GO) analysis (http://www.geneontology.org). *P* and *Q* values were used to test the reliability of the analysis. Kyoto Encyclopedia of Genes and Genomes (KEGG) pathway analysis (https://www.kegg.jp/) predicts potential biological functions and interactions between differentially expressed genes [32] (*P* < 0.05).

## 3. Statistical Analysis

SPSS version 24.0 (SPSS Inc., USA) was used to analyze the data in the study. The numerical data are presented as mean ± standard deviation (SD). Comparisons between the experimental group and the control group were performed using Student's *t*-test. The Pearson correlation index was used to calculate the correlation between two groups.

Results were considered statistically significant when *P* < 0.05.

## 4. Results

### 4.1. Transcriptome Sequencing

**Figure 1:**
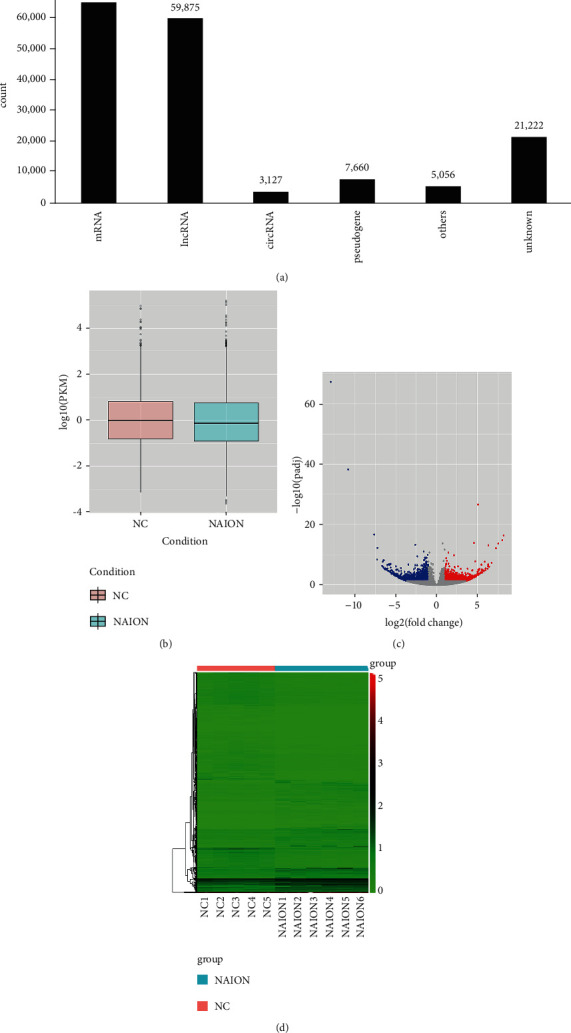
Transcriptome sequencing. (a) Total transcriptome expression types and counting histogram. (b) Box plot of transcript FPKM in NAION and NC. The vertical coordinate is log10 FPKM. Maximum, upper quartile, median, lower quartile, and minimum are listed from top to bottom. (c) Volcano map. The abscissa represents the log2 fold value of the fold difference in the expression of transcripts in both groups, and the ordinate represents the negative logarithm of the *P* value of the changes in transcripts. Red dots highlight upregulated transcripts, and blue dots reflect downregulated transcripts. (d) Cluster analysis. Red and green shadows, respectively, represent the levels above and below the relative expression in all samples. Cluster analysis was based on expression variation fold > 2.0 and *P* < 0.05.

High-throughput sequencing results showed that a total of 162,109 transcripts and 42,107 genes were detected. There were 3,127 circRNAs, 59,875 lncRNAs, 65,160 mRNAs, 5,056 other RNAs, 7,660 pseudogenes, and 21,222 unknown RNAs in the transcriptome (Figure 1(a)). By comparing the results of sequencing between the two groups, 3,255 transcripts and 1,406 genes were found differentially expressed in the two groups, of which 1,396 transcripts were upregulated and 1,859 were downregulated, while 630 genes were upregulated and 776 genes were downregulated, and the absolute value of log2 fold was >1 (*P* < 0.05). Nearly 46% of the expression of all transcripts was lower than 0.5 fragments per kilobase of per million mapped reads (FPKM), with about 0.6% above 50 FPKM (Figure 1(b)). The differential expression between NAION and controls can be described by the volcano map (Figure 1(c)) and cluster analysis (Figure 1(d)). In the volcano map, each point in the picture represents a transcript. The abscissa represents the log2 fold value of the fold difference in the expression of transcripts in both groups, and the ordinate represents the negative logarithm of the *P* value of the changes in transcripts. The larger the absolute value of abscissa, the greater the difference in expression between the two groups; the larger the ordinate value, the more significant the difference. Red dots highlight upregulated transcripts, and blue dots reflect downregulated transcripts. Hierarchical cluster analysis showed that transcripts were differentially expressed between NAION patients (NAION) and healthy controls (NC). Red and green shadows, respectively, represent the levels above and below the relative expression in all samples. Cluster analysis was based on expression variation fold > 2.0 and *P* < 0.05.

### 4.2. Differences in circRNA and mRNA Expression between Subjects with and without NAION

**Figure 2:**
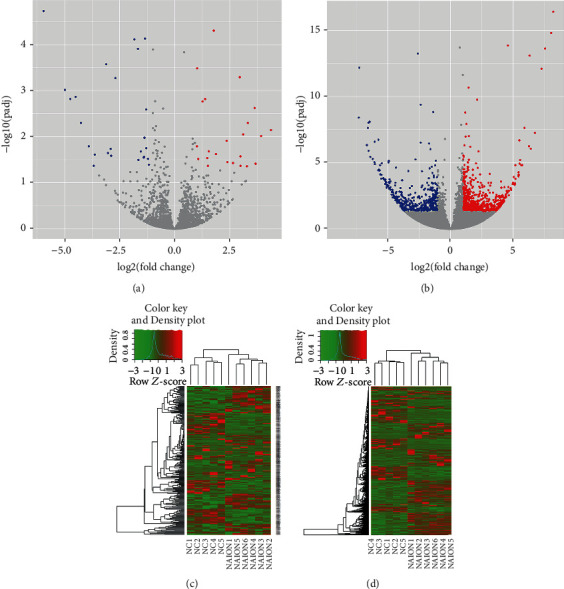
The volcano maps depict differentially expressed circRNAs (a) and mRNAs (b). Red dots highlight the upregulated circRNAs and mRNAs, and blue dots reflect downregulated circRNAs (c) and mRNAs (d). Hierarchical cluster analysis showed that circRNAs and mRNAs are differentially expressed between NAION patients (NAION) and healthy controls (NC). Red and green shadows, respectively, represent the levels above and below the relative expression in all samples. Cluster analysis was based on expression variation fold > 2.0 and *P* < 0.05.

A total of 3,127 circRNAs and 65,160 mRNAs were detected by high-throughput sequencing. Among them, 49 circRNAs and 1,161 mRNAs were dysregulated, including upregulation of 24 circRNAs and 698 mRNAs, while 25 circRNAs and 463 mRNAs were downregulated. The volcano maps were used to visually describe the differences between circRNAs and mRNAs from the two populations. Each point in the figure represents circRNAs and mRNAs (Figures 2(a) and 2(b)), and the abscissa represents the log2 fold value of the fold difference between circRNAs and mRNAs in the two groups, and the ordinate represents the negative logarithm of changed *P* value. The larger the absolute value of abscissa, the greater the difference of expression between the two groups; the larger the ordinate value, the more significant the differential expression. Then, cluster analysis was performed on differentially expressed circRNAs and mRNAs of the NAION group and the control group (Figures 2(c) and 2(d)). Cluster analysis was based on expression variation fold > 2.0 and *P* < 0.05.

### 4.3. Coexpression, PPI Network, and Module Analysis

**Figure 3:**
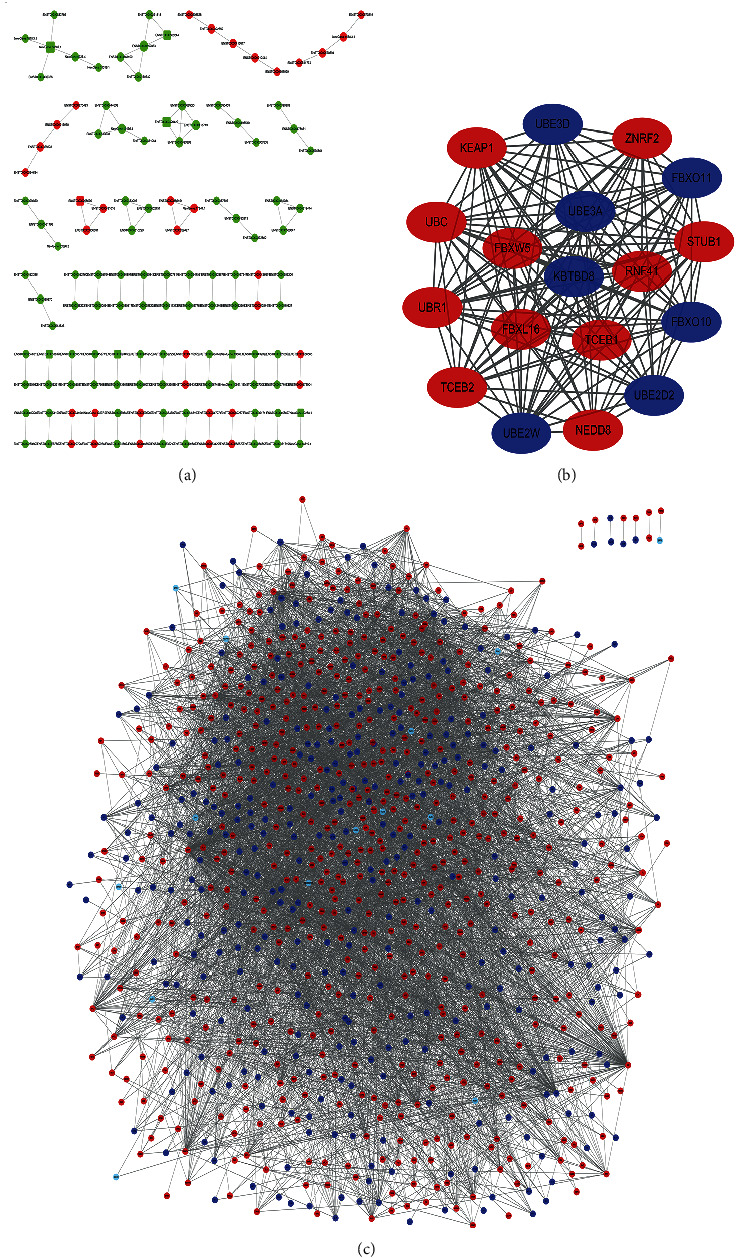
Differential genes' network. Coexpression, PPI network, and the most significant module of DEGs. (a) The absolute value of Pearson correlation coefficient was limited to bigger than 0.99 and *P* < 0.001. Red dots represent upregulated genes; green dots represent downregulated genes. “○” represents for mRNAs; “□” represents for circRNAs. The PPI network of DEGs was constructed by Cytoscape. DEGs were selected with a ∣fold change | >2 and *P* value <0.01 among the mRNA expression profiling. (c) The most significant module (b) was obtained from the PPI network with 18 nodes and 149 edges. Upregulated proteins are marked in light red; downregulated proteins are marked in light blue.

We constructed a coding-noncoding coexpression network by choosing significantly dysfunctional expressed transcript (Figure 3(a)). A total of 7 circRNAs and 121 mRNAs were included in the coexpression network. The network showed that these RNAs affect the pathological process of NAION in a variety of ways, including effects on metabolism, immune response, cell cycle, angiogenesis, and other biological processes. In the present study, the PPI network of 1,161 mRNAs was constructed using the STRING database, and an interaction with a combined score > 0.4 was considered statistically significant. The most significant module (Figure 3(b)) was obtained from the PPI network with 18 nodes and 149 edges, of which the hub gene is KBTBD8 (Kelch Repeat and BTB Domain-Containing 8), a protein-coding gene. Diseases associated with KBTBD8 include Treacher Collins Syndrome 1. Among its related pathways are the innate immune system and class I MHC-mediated antigen processing and presentation [33]. (Figure 3(c)).

### 4.4. GO Annotation and KEGG Analysis

**Figure 4:**
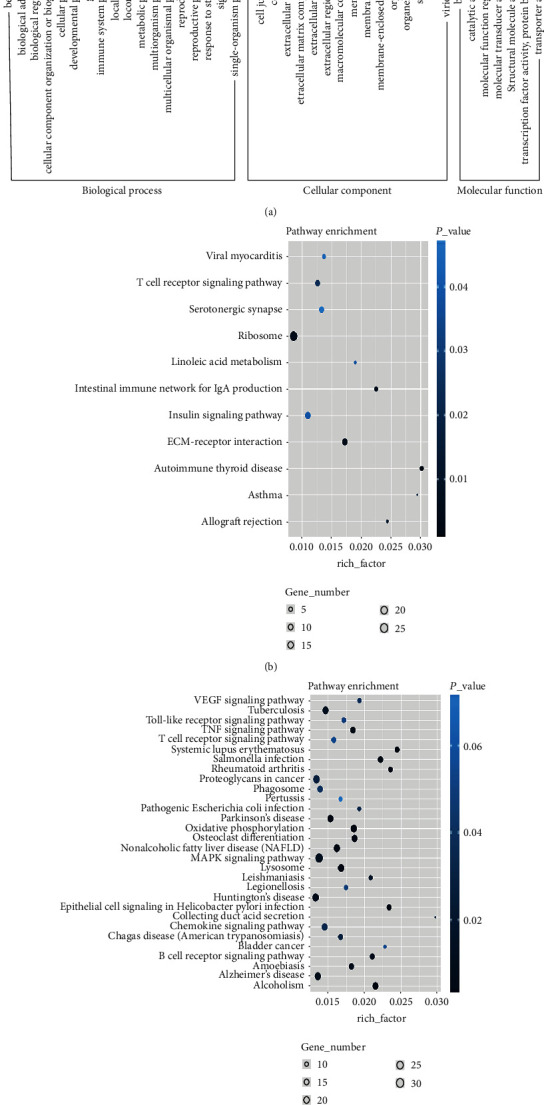
Analysis of GO and KEGG pathways of differentially expressed genes. GO and KEGG pathway analysis of differentially expressed genes. (a) GO annotation of upregulated and downregulated genes in biological processes, cell components, and molecular functions (*P* < 0.001 and *Q* < 0.05). The right *y*-axis represents the number of genes contained in the term; the left *y*-axis represents the percentage of genes. (b) and (c) rich bubble maps of downregulated and upregulated genes. Enrichment factors represent the ratio between the differentially expressed gene and all of the annotated genes rich in the pathway. Bubble scale represents the number of different genes; bubble color depth represents *P* value.

Ontology analysis of GO genes showed that a total of 587 enriched genes were upregulated, while 376 enriched genes were downregulated. These significantly enriched gene groups are shown in Figure 4(a), of which biological regulation, cellular processes, metabolic processes, and multicellular biological processes are the differential gene types that are significantly associated with biological process expression. Cells, cellular components, macroscopic molecular processes, and organelles are genes that are significantly associated with cellular components. Binding and catalytic activity are differentially expressed genes associated with molecular functions. At the same time, the KEGG pathway analysis revealed that these differentially expressed genes involved 52 upregulation pathways and 11 downregulation pathways as shown in Figures 4(b) and 4(c). Significant upregulation pathways in the upregulation pathways were found involving mitogen-activated protein kinase signaling pathways, tuberculosis, Huntington's disease, Alzheimer's disease, oxidative phosphorylation, oxidative phosphorylation, osteoclast differentiation, etc. Significantly downregulated pathways involve ribosomes, T cell receptor signaling pathways, extracellular matrix receptor interactions, and insulin signaling pathways. These clearly regulated signaling pathways involved in gene metabolism, neuronal degeneration, oxidative stress, and endocrine metabolism.

### 4.5. Identification of Differentially Expressed circRNAs by qRT-PCR

**Figure 5:**
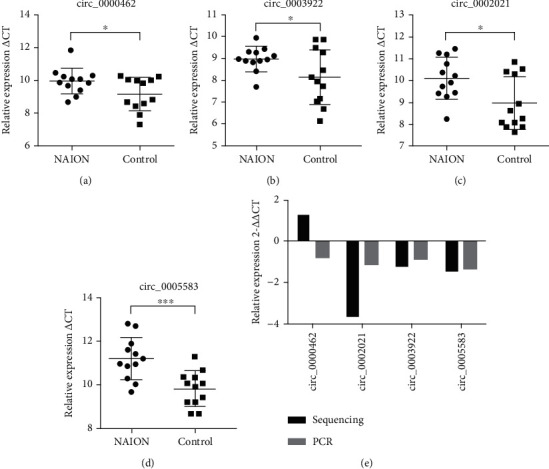
Screening for RNA expression detection. The qRT-PCR validation of selected RNAs. (a–d) The expression of selected RNAs was verified in 24 samples by qRT-PCR. The values were expressed as mean ± SD. (e) Comparison between qRT-PCR and sequencing results. The ordinate represents the mean variation fold (log2 scale) of each RNA, respectively, measured by qRT-PCR and sequencing. ^∗^Indicates *P* < 0.05 and ^∗∗∗^indicates *P* < 0.001.

qRT-PCR was performed in circRNAs with significant differences which were selected from the top ten significant genes, including 3 upregulated circRNAs and 7 downregulated circRNAs. circRNAs were predicted in circBase based on splicing length, and unlabeled circRNAs were labeled as new transcripts to be identified. We then performed qRT-PCR in 24 samples to verify the sequencing results. The results showed that the expression difference of the four circRNAs was statistically significant (*P* < 0.05). The verification results and sequencing results of the three circRNAs (hsa_circ_0003922, hsa_circ_0005583, and hsa_circ_0002021) are consistent, while the verification results of hsa_circ_0000462 are opposite to the sequencing results. And 4 circRNAs were downregulated in the NAION group. The results are listed in Figure 5.

## 5. Discussion

Nonarteritic anterior ischemic optic neuropathy (NAION) is a group of anterior optic nerve ischemic lesions caused by different etiology and pathogenesis. Our aim is to further predict the role of genes in the pathogenesis and prognosis of NAION by identifying differences in gene expression. The content of this paper has been automatically accepted in the postgraduate's thesis database of CNKI (China National Knowledge Infrastructure) [34]. In this study, we first used high-throughput sequencing and qRT-PCR to detect and validate differentially expressed circRNAs and mRNAs in the NAION group and the control group. Functions and roles of these genes are predicted by further analysis of sequencing and validating results based on existing biological analysis techniques. A total of 162,109 transcripts and 42,107 genes, 3,127 circRNAs, and 65,160 mRNAs were determined in this study, of which 49 circRNAs and 1,161 mRNAs were significantly dysregulated. We selected top 10 differentially expressed circRNAs for qRT-PCR validation. Further prediction and analysis of the sequencing results and verification results showed that these differentially expressed circRNAs might play an important role in the occurrence and development of NAION disease and act as important biomarkers.

There are 4 circRNAs with significant differences in the NAION group. They are hsa_circ_0005583, hsa_circ_0003922, hsa_circ_0002021, and hsa_circ_0000462.

In the results of this study, hsa_circ_0003922, hsa_circ_0005583, hsa_circ_0002021, and hsa_circ_0000462 are significantly downregulated (*P* < 0.05), especially hsa_circ_0005583 (*P* < 0.001). hsa_circ_0005583 was found closely related to the ataxia-telangiectasia mutated (ATM) gene. ATM gene mutations could cause various diseases such as ataxia-telangiectasia and increase sensitivity to cancer, diabetes, immunodeficiency, and exposure to ionizing radiation. All of these are characterized by neurodegeneration, chromosomal instability, immunodeficiency, and oxidative stress [35]. In the retina of ATM-deficient mice, glial fibrillary acidic protein (GFAP) staining showed morphological changes in astrocytes and impaired endothelial cell interaction leads to vascular leakage and decreased retinal function [36]. In addition, some researchers have explored the role of ATM in eye development and disease [37–39]. ATM protein is mainly located in the nucleus of retinal cells and nonneuronal cells of adult mice eyes. High levels of activated phosphorylated ATM (ATMp) were detected in the nucleus and cytoplasm of most ocular cell types. However, unlike other retinal cells, ATM immunostaining in photoreceptor cells seems to be confined to the nucleus, while ATMp immunostaining is confined to the cytoplasm. The specific patterns of ATM and ATMp immunoreactivity appear to be the same in mouse cerebellar granule cells as observed in mouse photoreceptor cells. This may indicate that these cells are particularly sensitive to oxidative damage [39]. This suggests that ATM and its phosphorylated activation forms may be involved in protecting cells from oxidative damage and maintaining the structure and function of ocular cells. In addition, early cataracts and retinal and choroidal vascular abnormalities (irradiated peripheral telangiectasia) were also found to be associated with ATM variation [40, 41].

As an extension of the central nervous system (CNS), the retina and optic nerve show many similarities with the brain and spinal cord in terms of anatomy, function, response to injury, and immunology [42]. Shen and his colleagues found that ATM signaling failure was associated with neuronal death in Alzheimer's disease (AD) [43]. Similarly, in our study, the KEGG pathway shows that circRNAs are closely related to pathways involved in neurodegenerative diseases such as AD and Huntington's disease. Our study found that these circRNAs also play important roles in both the optic nerve and the central nervous system, which requires further research and exploration.

hsa_circ_0003922, hsa_circ_0005583, hsa_circ_0002021, and hsa_circ_0000462 were predicted by the CircInteractome (Circular RNA Interactome: https://circinteractome.nia.nih.gov/index.html) database that their common RNA-binding protein (RBP) is the eukaryotic initiation factor 4A3 (eFI4A3) protein. eFI4A3 is a member of the DEAD-box RNA helicase family, while eFI4A3 mainly exists in the nucleus and has a high affinity with MAGOH-Y14. After binding with MAGOH-Y14, eFI4A3, together with metastatic lymph nodes 51 (MLN51), forms exon junction complex (EJC) which can bind tightly to newly generated mRNAs and participate in various subsequent splicing events, such as play an essential role in nucleation, subcellular localization, and translation of mRNAs [44]. Due to the low activity of the helicase for eFI4A3, it can participate in the localization of EJC in mRNAs as an RNA clamp [45]. Eukaryotes can eliminate the transcription products containing the premature stop codon by non-sense-mediated degradation of mRNAs, preventing the production of truncated proteins, which is a highly conserved and effective monitoring mechanism widely found in eukaryotes. eFI4A3 interacts with MAGOH and Y14 in MAGOH-Y14 and participates in the regulation of non-sense-mediated degradation of mRNAs [46], regulating the effects of harmful truncated proteins on the body.

The decrease of circRNA expression in this study may affect the quantity of RNA-RNA-binding protein complex and thus affect the expression of related proteins. It may be involved in the non-sense-mediated degradation of mRNAs in the pathogenesis of NAION, leading to the expression variation of optic nerve-related proteins. It may also participate in the antioxidant process in vivo by regulating the synthesis of selenium protein and directly or indirectly leading to apoptosis, and the defects of protein synthesis in the neuroregulatory region cause nerve damage in the regulatory region.

The hsa_circ_0000462 sequencing result was opposite to the qRT-PCR verification result. The same sequencing backup sample was used again for qRT-PCR verification, and the result of the second verification was consistent with the first one. The inconsistency may be due to the differences between the two technology platforms.

The following problems also exist in our study: Due to the restriction of anterior chamber/vitreous puncture and biopsy of the optic nerve and retina in NAION patients, this study only detected the expression of RNAs in peripheral blood, but not in the retina and optic nerve, which are closely related to the optic nerve. All the experimental results are only prediction results. We will further carry out cell model experiments to conduct function and localization studies on abnormally expressed genes, which will help us comprehend the relationship between NAION and differential genes more deeply.

In conclusion, sequencing analysis provides differential expression profiles of RNAs, especially circRNAs and mRNAs. Differentially expressed circRNAs may provide new ideas for the etiology of nonarteritic anterior ischemic optic neuropathy and provide basic information for subsequent research.

## Figures and Tables

**Table 1:** Basic situation of the experimental group and the control group.

	NAION (*n* = 18)	Control (*n* = 17)	*P* value
Age (years)	65.780 ± 2.365	64.530 ± 1.663	0.082
Sex, female/male	9/9	9/8	1.000
Mean ± standard deviation age of onset (days) (M + SD)	9.500 ± 3.884	0.000	—
Fasting blood sugar (mmol/L)	5.306 ± 0.162	5.311 ± 0.117	0.917
Glycated hemoglobin (%)	4.594 ± 0.398	4.606 ± 0.441	0.936
Cholesterol (mmol/L)	4.077 ± 0.273	4.178 ± 0.254	0.264
Triglyceride (mmol/L)	0.939 ± 0.150	0.939 ± 0.109	0.999
High-density lipoprotein (mmol/L)	1.333 ± 0.145	1.308 ± 0.177	0.656
Low-density lipoprotein (mmol/L)	2.587 ± 0.536	2.795 ± 0.517	0.252
Systolic pressure (mmHg)	118.944 ± 4.304	119.294 ± 5.022	0.826
Diastolic pressure (mmHg)	77.389 ± 3.728	78.647 ± 4.167	0.353

**Table 2:** Primer sequences of circRNAs for qRT-PCR validation.

Gene name	Test gene	Primer	Sequence (5′-3′)
ANKIB1	hsa_circ_0001723	Forward primer	GCCCTCAATCTGGAATCTCA
		Reverse primer	CTTCAGCCACTTCTGGAACA
SLC15A4	hsa_circ_0000462	Forward primer	AGTGGAGAGCGCCAGAGTAA
		Reverse primer	ATAGGCAATGCCACCTAACG
STAU2	hsa_circ_0001811	Forward primer	GGAGAGCCTGCCATCTACAG
		Reverse primer	GATTCCCATGTCTGCTCACC
SP100	hsa_circ_0003922	Forward primer	CAACAGAGTCCTGCGAACAA
		Reverse primer	GAGCAGCCTGTCATCTACACC
EIF4E3	hsa_circ_0066536	Forward primer	GTGGGTGAAGCGACTGTTTT
		Reverse primer	CACTCCAAAATATCTGTACTGTCTG
ATM	hsa_circ_0005583	Forward primer	TCACCAGCTGTCTTCGACAC
		Reverse primer	TGTCCAGTCTTTGTGGCTAAA
BACH2	hsa_circ_0001626	Forward primer	AAAGTGAGGGCTCCAGGAAT
		Reverse primer	GCTTGGTCCCAAATGATGTC
RAB6A	hsa_circ_0000339	Forward primer	GAGGAAAGCCAAAGAGCTGA
		Reverse primer	CCACAGTGGAGTCACGAATG
CLNS1A	hsa_circ_0023694	Forward primer	ATGAAGATGGGATGGAGGTG
		Reverse primer	TTCCTCTTCTTCATCAGCAACA
FRYL	hsa_circ_0002021	Forward primer	TCCTGAAACCACTCAAGAGTCA
		Reverse primer	GGCACACTGGTACAAAGCAG

## Data Availability

The sequencing profile data used to support the findings of this study are included within the supplementary information file(s) uploaded to the system.
